# Beneficial effects of bioinspired silver nanoparticles on zebrafish embryos including a gene expression study

**DOI:** 10.5599/admet.2102

**Published:** 2024-01-01

**Authors:** Sakthi Devi R, Agnishwar Girigoswami, Shanmugaraja Meenakshi, Balasubramanian Deepika, Karthick Harini, Pemula Gowtham, Pragya Pallavi, Koyeli Girigoswami

**Affiliations:** Medical Bionanotechnology, Faculty of Allied Health Sciences, Chettinad Hospital & Research Institute (CHRI), Chettinad Academy of Research and Education (CARE), Kelambakkam, Chennai-603 103, India

**Keywords:** Green synthesis, biocompatibility, nanoparticle toxicity, fish embryo, zebrafish hatching (ZHE1 and ZHE2)

## Abstract

**Background and purpose:**

Many sectors use nanoparticles and dispose of them in the aquatic environment without deciding the fate of these particles.

**Experimental approach:**

To identify a benign species of nanoparticles which can cause minimum harm to the aquatic environment, a comparative study was done with chemically synthesized silver nanoparticles (AgNPs) and green tea mediated synthesis (GT/AgNP) in both in vitro using human alveolar cancer cell line (A549) and normal cell line (L132), and in in vivo with zebrafish embryos.

**Key results:**

The in vitro studies revealed that GT/AgNPs were less toxic to normal cells than cancer cells. The GT/AgNPs showed high biocompatibility for zebrafish embryos monitored microscopically for their developmental stages and by cumulative hatchability studies. The reduced hatchability found in the AgNPs-treated group was correlated by differential gene expression of zebrafish hatching enzymes (ZHE) (ZHE1 and ZHE2).

**Conclusion:**

The results indicated that nanoparticles can affect the hatching of zebrafish embryos and elicit toxicity at the gene level.

## Introduction

Over the decennium, research in nanomaterials has advanced and even revolutionized various fields like biological, pharmacology, biomedical, chemical sciences, food safety, diagnostics, information technology, environmental sciences, agriculture, *etc.* [1]. The nanoparticles (NPs) are available in different shapes (triangular, rod, polyhedral, round, octagonal) and amid the various NPs, metal and metal oxide-based NPs have been explored extensively due to their characteristic property. The application of these metal NPs is further primitive compared to other nanostructures. Silver nanoparticles (AgNPs) were utilized as a medicine or component of medicine in India and China for the preparation of ayurvedic or natural medicine [2]. AgNPs were mostly used in all durables, like textiles, cosmetics, and medical equipment like catheters. This paved the way for the environment and humans to get exposed to the AgNPs [3]. It also played a role in disinfecting medical equipment and treating water. In 2015, it was reported that there was a massive production of more than 550 t globally of AgNPs, using 450 products. It is expected that by 2025, the production rate will be increased to 800 t due to the development of the nanotechnology industry. Universally, each year, the load of AgNPs that enters the waterbodies is approximately 63 t, which means an accumulation of approximately 40-80,000 μg/kg in sediments and 10-1800 ng/L in water surfaces [4]. AgNPs are used mostly in daily products and also for therapeutic purposes. Due to the interaction of AgNPs with the organic matter, oxygen, cations, and different pH, their properties alter and finally release toxic Ag^+^ ions. This causes environmental toxicity. The use of AgNPs, which get disposed of in the aquatic system, increases day by day. There are several negative impacts on AgNPs exposure. Many studies have demonstrated the biological impact and the impact of AgNPs in crossing the chorionic pores. Some metal nanoparticles can pass through the chorionic pores and result in unforeseeable effects. Nanomaterial synthesis also plays a crucial role in biology. AgNPs are widely synthesized by chemical reduction method using borohydrate or sodium citrate. As they are more toxic, eco-friendly, and biodegradable, green synthesis has come into existence [5], exerting safe effects [6].

Zebrafish is a promising model for evaluating developmental abnormalities after exposure to different toxicants [7]. The hatching process is one of the pivotal steps in the development of zebrafish, and the hatching glands formation and movement are responsible for the synthesis and secretion of the hatching enzymes [8]. The process of hatching of zebrafish embryos consists of two steps. First, the hatching enzymes break down the inner radiation zone of acellular chorion secreted by the hatching glands. Finally, the weakened egg chorion is broken by the impulsive movement of embryos [9, 10]. Three orthologues of zebrafish hatching enzyme (ZHE), namely ZHE1a, ZHE1b and ZHE2, are clustered in the genome according to the zebrafish genome project. There is a wide range of amino acid sequence similarity between genes, such as the ZHE1a sequence, which is 99 % similar to ZHE1b and 60.8% similar to that of ZHE2 [9]. There is a lack of previous studies on the effect of AgNPs up to the hatching enzyme gene expression level in zebrafish embryos. The current study explores the toxic effect of chemically synthesized silver nanoparticles (AgNPs) and green tea-mediated silver nanoparticles (GT/AgNPs) in both *in vitro* using cell lines and *in vivo* in zebrafish model organisms at very low concentrations. The study focused on low nanoparticle concentration because the size and concentration of any nanoparticle play a pivotal role in inducing toxicity. The *in vitro* toxicity was analysed in both cancer cells and normal cells using different methods, such as, MTT assay, live dead assay using fluorescence microscopy, and scanning electron microscopy (SEM) imaging. Further, the *in vivo* analysis was done using zebrafish embryos monitoring the embryo development microscopically as well as by observing the cumulative hatchability. Finally, the hatching genes (ZHE1 and ZHE 2) expressions were also studied in AgNP and GT/AgNP treated embryos.

## Experimental

### Materials

Silver nitrate was purchased from Rankem brand, India. The Hi-cDNA synthesis kit (MBT076-10R) was purchased from HiMedia, the master mix for PCR from Thermofisher Scientific and the specific primers for the gene ZHE1 & ZHE2, beta-actin (Sigma Aldrich) from BioSource & Surgical. The A549 cell line and L132 cell line were purchased from NCCS, Pune. Dulbecco’s Modified Eagle Medium (DMEM), antibiotic solution, diethylpyrocarbonate (DEPC), MTT (3-(4,5-dimethylthiazol-2-yl)-2,5-diphenyltetrazolium bromide) was from HiMedia; bovine serum (FBS) was purchased from Gibco; dimethyl sulphoxide, acridine orange (AO), ethidium bromide (EtBr), phosphate-buffered saline (PBS) pellets were purchased from Rankem brand, India. Green tea was purchased from Korakundah Estate.

### Synthesis of chemical-mediated silver nanoparticles

AgNPs were synthesized by the chemical reduction method described earlier [11] using trisodium citrate as a reducing agent. In 100 ml of distilled water, 25 ml of aqueous 5mM of Silver nitrate solution was added and boiled to heat. 5 ml of trisodium citrate solution (1 %) was quickly added to the boiling solution. Heating was continued until the colour changed to light yellow, followed by stirring. The working concentration of AgNPs was 1 mM, diluted for further studies using water (for zebrafish embryo study) and complete DMEM (for cell culture studies).

### Synthesis of green tea-mediated silver nanoparticles

An amount of 2 g of green tea was soaked overnight in 100 ml of distilled water and in another beaker, 10 mM of silver nitrate solution was prepared. 0.5 ml of 10 mM of silver nitrate solution was taken and 0.4 ml of tea extract was added. Further, 9 ml of distilled water was added and kept at room temperature until the colour changed to dark brown, indicating the formation of silver nanoparticles. The working concentration of GT/AgNPs was 0.5 mM, diluted for further studies using water (for zebrafish embryo study) and complete DMEM (for cell culture studies).

### Characterization of synthesized nanoparticles

The as-synthesized AgNPs and GT/AgNPs were characterized using different photophysical tools as described earlier [12]. The nanoparticle samples were diluted in distilled water and proceeded to measure the absorption spectrum using a Shimadzu UV-1800 absorption spectrophotometer. Further, to measure the particle size of the nanoparticle and surface charge distribution, a Malvern nano ZS90 zeta sizer instrument was used. The scattered intensity data was processed in the instrumental software, and the hydrodynamic diameter (nm) and surface charge were obtained. Bruker alpha FT-IR spectrometer was used in the transmittance mode to record the FTIR spectra, which infers the chemical bonds and their stretching, bending vibrations, etc., present in the compound. The samples were liquid, so the attenuated reflectance (ATR) mode was used to record the transmittance percentage with respect to the wavenumber in cm^-1^.

### Antimicrobial activity of the synthesized AgNPs and GT/AgNPs

Since the study investigates the low-concentration effect of silver nanoparticles, it is important to know whether, at this concentration, the synthesized AgNPs could exert any antimicrobial activity or not [13]. The overnight grown culture of the laboratory *E. coli* K12 strain MG1655 was sub-cultured in 2 ml of freshly prepared LB broth taken in four different test tubes labelled as T1, C1, T2, C2 (T1-AgNPs, C1-reducing agent trisodium citrate, T2-GT/ NPs and C2-reducing agent green tea) and allowed to grow aerobically for about 90 min at 37 °C. When the culture reached the mid-log phase, the NPs (0.2 nM) and reducing agents (equal volume to the NPs solution) were added to the respective tubes and incubated again. Around 0.1 ml of the culture was withdrawn at regular intervals (0, 3 and 6 h), serially diluted up to 10^-6^ in saline. From each dilution, 0.01 ml was taken and sequentially spotted on LB agar plates. The plates were incubated at 37 °C for about 24-36 h, the colonies were counted and the cfu/ml was calculated. The antibacterial activity was assessed based on the colony-forming unit.

### In vitro cytotoxicity testing using A549 and L132 cells by MTT assay

Two types of cells, A549 (lung cancer cells) and normal cells (L132) were used to study the toxicity of the synthesized AgNPs and GT/AgNPs at low concentrations. To determine the cell viability, an MTT assay was done according to Metkar et al. 2022 [14]; in brief, in a 24-well plate, 1.15×10^4^ of A549 and 1.4×10^4^ L132 cells, respectively, were grown in DMEM with 10 % FBS and antibiotics (1%) supplementation. The plate was incubated for 24 h at 37 °C in a humidified atmosphere with 5 % carbon dioxide. Then, both A549 and L132 cells were treated with AgNPs and GT/AgNPs at the doses of 0.05, 0.1, 0.2, 0.3 and 0.5 nM, respectively. After 24 h of treatment, 25 μl of MTT (10 mg/ml) was added to each well in dark condition and kept for 4 h incubation at 37 °C in a CO_2_ incubator for the development of formazan crystals. Further, 1 ml of DMSO was added to each well and mixed properly to solubilize the formazan crystals formed and then the optical density (O.D.) was noted at 570 nm. The cell viability (%) was then calculated using Equation (1):



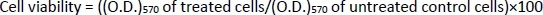

(1)


The experiment was repeated thrice.

### Examination of live and dead cells using fluorescent microscopy

Since the results of the MTT assay showed that the GT/AgNPs killed specifically the cancer cells, not the normal cells, the live dead assay using EtBr and AO was executed only for the cancer cells, according to Liu *et al.* [15], with slight modification. The live cells will be green in colour, whereas the dead cells will take red colour under a fluorescent microscope. The exponentially growing A549 cells were grown on a coverslip and were treated with AgNP and GT/AgNP at a dose of 0.2 nM (the dose was decided based on MTT assay results). After treatment, plates containing the coverslips were incubated for 24 h. For live dead assay, 1.2 mM of AO and 1.9 mM of EtBr were prepared. The working solution (dye mixture) was prepared by dissolving 5 μl of AO and 2 μl of EtBr in 2 ml of PBS in the dark. The coverslips containing the treated and untreated cells were incubated with the dye solution at 37 °C in the dark for 3 min and then visualized under a fluorescent microscope (Olympus, BX 51). The images were captured at different locations on the slide and the number of live and dead cells were plotted for both nanoparticle treatments. The experiment was done in triplicates and repeated two times.

### Scanning electron microscopy of cells treated with AgNPs and GT/AgNPs

The SEM analysis was done according to Girigoswami *et al.* [16]. The A549 cells were grown in glass coverslips placed inside petri plates for 24 h at 37 °C in a CO_2_ incubator. The cells are treated with 0.2 nM concentration of AgNPs and GT/AgNPs. 24 h after treatment, the cells were washed with PBS (pH 7.4) and then fixed with acetomethanol (acetone: methanol= 1:1) at 4 °C for 4 h. The fixing solution was thoroughly evanesced. Using SCD005Pt-coater (Bal- Tec AGI, Liechtenstein), samples were coated with a thin film of platinum. With the S-4800 scanning electron microscope (Hitachi High Technologies Co., Japan), electron micrographs were obtained with an acceleration voltage between 1 and 5 kV. To avoid the sample burning, higher acceleration voltages were not used [17].

### Zebrafish embryo toxicity study

The wild-type female and male zebrafish (*Danio rerio*) were cultured in separate rectangular glass tanks and the adult fishes were maintained at 25 ± 2 °C with a water pH 7.0. Air pumps were attached inside the fish tank for proper air circulation for the fish. Live blood worms and standard flakes were given to the fish twice a day. For spawning, the male and female fish of 1:2 ratio were kept in the breeding tank with 12 h light-dark condition and the next day, the embryos were collected and washed in water three to four times. Animal protocols were approved by the Committee for the Purpose of Control and Supervision of Experiments on Animals (CPCSEA) 2021, Department of Animal Husbandry and Dairying, Government of India. The Institute Animal Ethics Committee (IAEC) of Chettinad Academy of Research and Education approved the *Danio rerio* embryos (letter No. IAEC1/Proposal:81/A.Lr:61/Dt. 11.05.2022).

Cumulative hatchability was evaluated following our previous report [17]; in brief, to assess the toxicity, in a 6 well plate, zebrafish embryos were treated with different concentrations of AgNPs and GT/AgNPs (0.05, 0.1, 0.2, 0.3 and 0.5 nM). Each well contained 30 embryos and 3 ml of E3 medium. In a separate well of another 24-well plate, the control embryo was maintained under the same conditions containing only E3 medium. The embryos were observed under a compound microscope at different hours of post-fertilization (hpf) (10, 28, 53, 60, 77, 100, 124 and 148 hpf) and the images were captured accordingly. The cumulative hatchability was calculated with the formula for the control groups (Eq. (2)) and the different nanoparticle-treated groups:



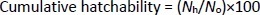

(2)


where, *N*_h_ is the number of eggs hatched and *N*_0_ is the total number of eggs taken for treatment with nanoparticles [17]. A cumulative hatchability graph was plotted, taking the time along the abscissa and the percentage of embryos hatched along the ordinate for different concentrations. The experiment was repeated three times.

### Gene expression study of ZHE 1 and ZHE2

The embryos were collected in a similar way as described above for extracting the mRNA, which was converted to cDNA and taken as a template for gene analysis, according to Ghosh and Girigoswami [17] and Peterson and Freeman [18,19], with a few modifications. Briefly, at 32 hpf, 40 embryos were exposed to 0.1 nM concentration of AgNPs and GT/AgNPs, respectively, and after 24 h, the embryos were taken for RNA isolation. Control embryos were maintained only in the E3 medium without any treatment.

### RNA isolation

After treatment, the RNA was isolated according to Ghosh and Girigoswami [17] and Peterson and Freeman [18,19], with some modifications. All the glassware and plasticware were soaked in DEPC water overnight, dried and autoclaved before the initiation of RNA isolation. Briefly, from each group, the embryos were taken in separate vial and homogenized on ice after adding 100 μl of Trizol reagent. Further, 0.9 ml of Trizol was added to the tubes individually and kept for 5 min at room temperature. Chloroform (0.2 ml) was added to these tubes and mixed vigorously several times, followed by 3 min incubation at room temperature. The mixtures were centrifuged at 12000 rpm at 4 °C for 15 min. The aqueous phase in the top layer was transferred into a fresh tube without disturbing the middle layer. To precipitate the RNA, 0.5 ml of isopropanol was poured into each tube, incubated for 10 min at room temperature and centrifuged at 12000 rpm for 5 min at 4 °C. Aspiration of the supernatant was done carefully without agitating the pellet. Washing of the pellet was done with 75 % ethanol using centrifugation at 12000 rpm at 4 °C for 5 min. Ethanol was removed after centrifugation, air-dried for 10 min and dissolved in RNase-free water. The RNA concentration was calculated by measuring the O.D. at 260 nm, and the RNA purity was calculated by the ratio of O.D.s at 260 nm and 280 nm. If the ratio was above 1.8, it was processed for cDNA synthesis.

### Preparation of cDNA

After RNA isolation, cDNA was prepared using a Hi-cDNA synthesizing kit (MBT076-10R) supplied by HiMedia. The following reagents were mixed as follows: in a RNase free PCR tube - 1 μl of Oligo (dT), 1 μl of random hexamer, 2 μl of (Random Hexamer: Oligo (dT) mix), 0.28 μg, 1.1 μg and 0.17 μg concentration of control, AgNP and GT/AgNP RNA sample was added respectively and made up to 10 μl with PCR water (molecular biology grade PCR water). The tubes were incubated for 5 min at 65 °C and immediately cooled in ice. To the above tube, the following reactants were added: RT buffer (M-MuLV) of 4 μl, 2 μl of 10X solution for M-MuLV, 1 μl of M-MuLV reverse transcriptase (RNase H), 0.5 μl of ribonuclease inhibitor, 2 μl of 10 mM of dNTP mix and finally made up to 20 μl with PCR water. Then, the tubes were mixed nicely and kept at 42 °C for 60 min, followed by 70 °C for 5 min (1 cycle). The tube was kept at 4 °C. The RT product was stored at -20 °C.

### PCR product for ZHE1 and ZHE2

The cDNA was successfully synthesized and was utilized to study the expression of ZHE1 and ZHE2 by PCR. The housekeeping gene, β actin, was taken as control. The sequences of primer used for amplification are as follows: β actin F: 5’-CGAGCTGTCTTCCCATCCA 3’; β actin R: 5’-TCACCAACGTAGCTGTCTTTCTG 3’.; ZHE1 F: 5’-CTGAACTTCTCTACACACTGAGG-3’; ZHE1 R 5’-CCTTATCACC ATCACCTCACTTC-3’; ZHE2 F: 5’-CTCCACACACTGAGACTAAATGG-3’; ZHE2 R: 5’-GGAAATAAGAGCACGTACTGTGG-3’. The reagents for PCR were added and mixed for each 0.5 ml tube as follows: 12.5 μl of 2X Master mix, which contains 0.05 U / μl of Taq DNA polymerase, 4 mM MgCl_2_, reaction buffer, and 0.4 mM of each dNTP. Then 0.5 μl of forward primer and reverse primer of a specific gene, 2.0 μl of template and 9.5 μl of molecular grade water were added. All the genes had common PCR condition: the initial denaturation was at 95 °C for 5 min, and then 35 cycles were run with each cycle containing the three steps: 30 s denaturation at 95 °C, 30 s annealing at 54 °C, and 45 s extension at 72 °C. After the 35 cycles, the final extension at 72 °C for 5 min was kept. The RT product was kept at 4 °C for 5 min and then stored at -20 °C.

### Visualization of the PCR product by agarose gel electrophoresis

Agarose gel (1 %) was prepared with tris acetate EDTA buffer, to which 4 μl of ethidium bromide was added and dissolved correctly. The gel boat was sealed and the gel was poured and allowed to solidify. 10 μl of each PCR sample was mixed with 5 μl of sample loading dye. The samples were loaded in their respective wells, and the gel was run at 100 V and visualized under a UV-transilluminator. The images were captured using Genei capture imaging software in a Gel-Doc (GeNei Imaging System). The intensity of the bands was calculated using Image J software.

## Results

### Characterization of the chemical and green synthesized silver nanoparticles

Silver nanoparticles synthesized via the green route were observed by a colour change after adding green tea extract (Figure S1). The characterizations of the AgNPs and GT/AgNPs were done using different photophysical tools for measuring their absorption spectrum, hydrodynamic diameter, surface charge and functional groups. Figure S2 shows the absorption spectrum of AgNPs and GT/AgNPs. The *λ*_max_ was found to be 429 nm for AgNP and 434 nm for GT/AgNP, which corroborates with the characteristic absorption peak of silver nanoparticles. AgNP’s and GT/AgNPs’ hydrodynamic diameters and zeta potentials are given in Figure S3. The hydrodynamic diameter obtained for AgNP was 65.39 nm and for GT/AgNP it was 106.14 nm. AgNP showed a surface charge of -25.1 mV and GT/AgNP was about -16.9 mV. The characteristic FTIR peaks obtained were noted. The visible peaks are present at 3312 cm^-1^ for O-H stretching, 1634 cm^-1^ for N-H bending, 664 cm^-1^ and 530 cm^-1^ for C-Br stretching for AgNP. For GT/AgNP, visible peaks obtained at 3319 cm^-1^, 1634 cm^-1^, and 538 cm^-1^ represent that O-H stretch, N-H bend, and C-Br stretch were present, respectively [20]. The FTIR spectra for AgNP and GT/AgNP are given in Figure S4.

### Anti-bacterial activity of the nanoparticles

Figure S5 shows the bacterial growth in AgNPs (0.2 nM) and GT/AgNPs (0.2 nM), as well as in the plates treated with reducing agents (trisodium citrate and green tea extract). There was no inhibition of *E. coli* growth after treatment with such a low dose of silver nanoparticles.

### In vitro cytotoxicity test using A549 and L132 cells

**Figure 1.**
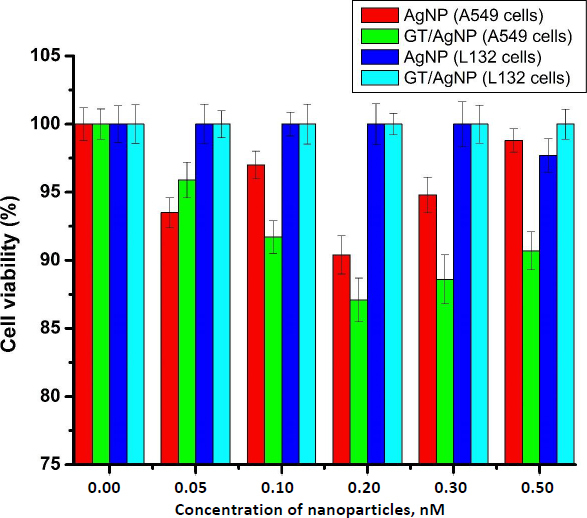
In vitro cytotoxicity analysis of A549 cells and L132 cells using MTT assay

The main focus of our study was to observe the effect of silver nanoparticles at very low doses. Cell viability of A549 cells and L132 cells after treatment with AgNP and GT/AgNP was assessed using an MTT assay. It was evident from the result that 0.2 nM concentration of GT/AgNP reduced the viability of A549 cells more efficiently in comparison to other concentrations. It was apparent from the result that neither chemically synthesized nor green tea-mediated synthesis affected the viability of L132 cells at different concentrations used. The comparative data is given in the graph below (Figure 1).

### Examination of live and dead cells using fluorescent microscopy

**Figure 2.**
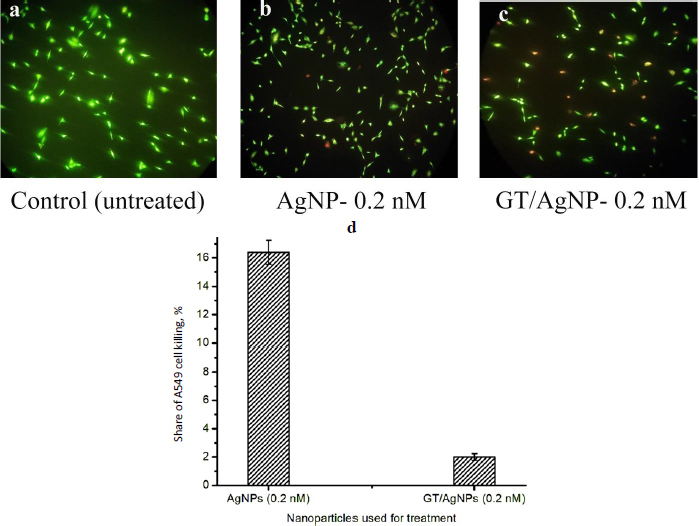
Typical fluorescence microscopic images of: a- control, b - AgNPs and c - GT/AgNPs treated A549 cells for live and dead cell assay, d - share of dead A549 cells as scored by live and dead cell assay

Live cells will appear green and dead cells red when visualized under a fluorescent microscope after staining with EtBr and AO. The typical snapshots of the cells viewed under a fluorescence microscope at 10 X magnification, 24 h after treatment with 0.2 nM AgNPs (Figure 2 (b)) and GT/AgNPs are shown in Figure 2(c). Control cells are shown in Figure 2(a). Exploring the live dead cell assay, the A549 cell killing efficiency of GT/AgNPs was found to be 16.4 % and for AgNPs, it was 2 %, respectively. The results were obtained by counting the live cells’ and dead cells’ numbers for the snapshots captured at different locations of the coverslip. The cell counting results showed the percentage of killed cells after treatment with the nanoparticles and are depicted in Figure 2(d). The comparative data infers that GT/AgNPs killed the cancer cells at a higher rate compared to the AgNPs.

### Scanning electron microscopy

**Figure 3.**
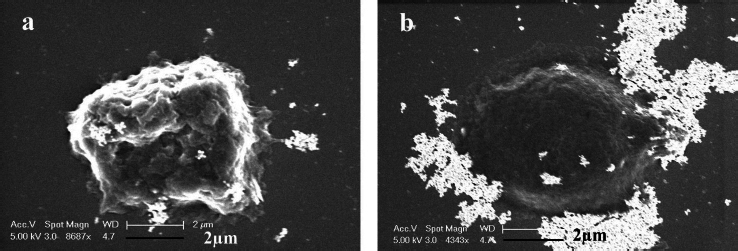
a - SEM image of A549 cells treated with GT/AgNP, b - SEM image of A549 cells treated with chemically synthesized AgNPs

To gain further insights into the size of the nanoparticles and characteristics of cancer cells after exposure to AGNPs and GT/AgNPs, SEM micrographs were taken. Figure 3 (a) and (b) show the SEM images of the A549 cells treated with GT/AgNPs (Fig 3 (a)) and chemically synthesized AgNPs (Fig 3 (b)). The typical cell shrinkage, which indicates apoptosis, was observed for GT/ AgNPs treated cancer cells, whereas chemically synthesized AgNPs treated cells did not show any toxicity to the cancer cells.

### Toxicity of zebrafish embryo and cumulative hatchability

**Figure 4.**
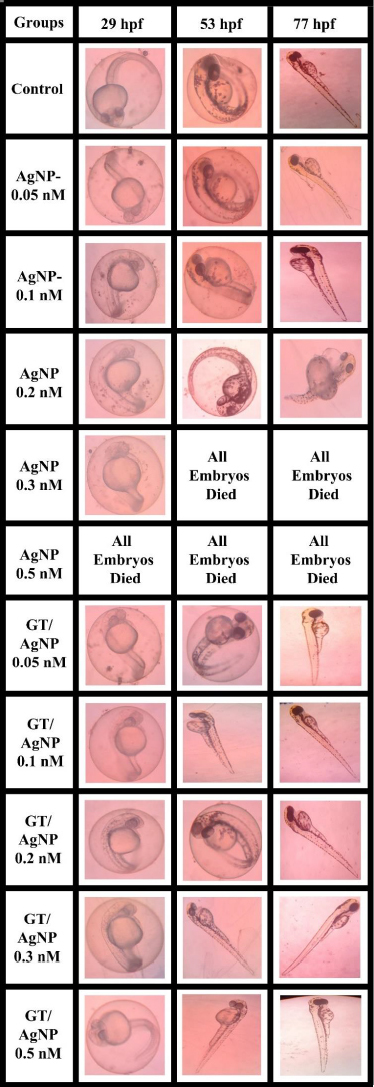
Comparative microscopic images for zebrafish embryo toxicity at different concentrations

**Figure 5.**
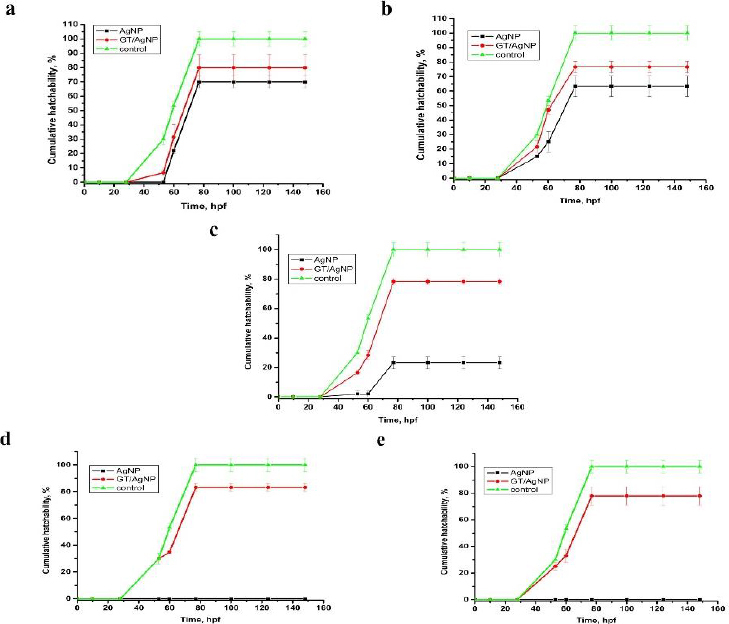
The cumulative hatchability of AgNP and GT/AgNP at concentrations: a - 0.05, b - 0.10, c - 0.20, d - 0.30 and e - 0.50 nM

The zebrafish embryos’ morphology was observed for control and after treatment with AgNPs and GT/AgNPs at different concentrations (0.05, 0.1, 0.2, 0.3 and 0.5 nM) (Figure 4). Depending on the embryo’s hatchability at different concentrations, cumulative hatchability was calculated and depicted in Figure 5. For 0.05 nM concentration, the AgNPs-treated embryos showed 70 % hatchability, whereas GT/AgNP-treated embryos showed 80 % hatchability. Abnormalities such as body bends were observed in only AgNPs-treated embryos. For 0.1 nM concentration, the hatchability percentage was 63 % for AgNPs and for GT/AgNP-treated embryos, it was 77 %, respectively. No delayed hatching was observed in these concentrations (0.05 and 0.1 nM).

**Figure 6.**
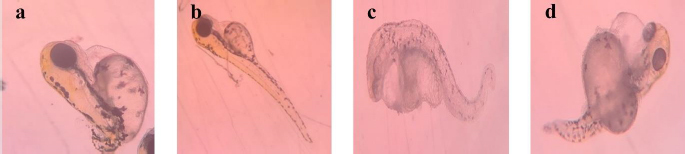
Developmental abnormalities of zebrafish embryos treated with 0.05 nM of AgNPs showed abnormal growth: a - pericardial oedema and b - tail bend. Embryos treated with c - 0.1 and d - 0.2 nM AgNPs, showed abnormal growth and tail bend

For 0.2 nM concentration, in chemical-AgNPs treated embryos, only 23 % of embryos hatched at 77 hpf with abnormalities like tail bend and 78 % at GT/AgNPs treated embryos, without showing any abnormality. The hatchability percentage decreased with increasing concentrations of AgNPs-treated embryos. For 0.3 nM concentration, all the AgNPs treated embryos were dead, whereas GT/AgNPs treated embryos showed 83 % hatchability. 0.3 nM concentration of GT/AgNP showed an increased hatchability percentage in comparison to other concentrations. For 0.5 nM concentration, all embryos died in AgNPs-treated embryos and in GT/AgNPs-treated embryos, it showed 78 % of hatching and there was no delayed hatching found. The highest hatchability of 83 % was observed in GT/AgNP of 0.3 nM concentration. On the other hand, all the embryos treated with higher concentrations of AgNPs, such as 0.3 and 0.5 nM, died. Abnormalities like body bend, abnormal growth, and tail bend were seen in embryos treated with AgNP of 0.05 nM, 0.1 nM, and 0.2 nM alone, whereas GT/AgNP-treated embryos showed no developmental defects microscopically (Figure 6).

This study confirmed that biosynthesized silver nanoparticles using green tea (GT/AgNP) have reduced toxicity in zebrafish embryos compared to AgNP-treated embryos. The microscopic images of chemically synthesized treated embryos with developmental disabilities are shown in Figure 6.

### Gene expression of ZHE1 and ZHE2

**Figure 7.**
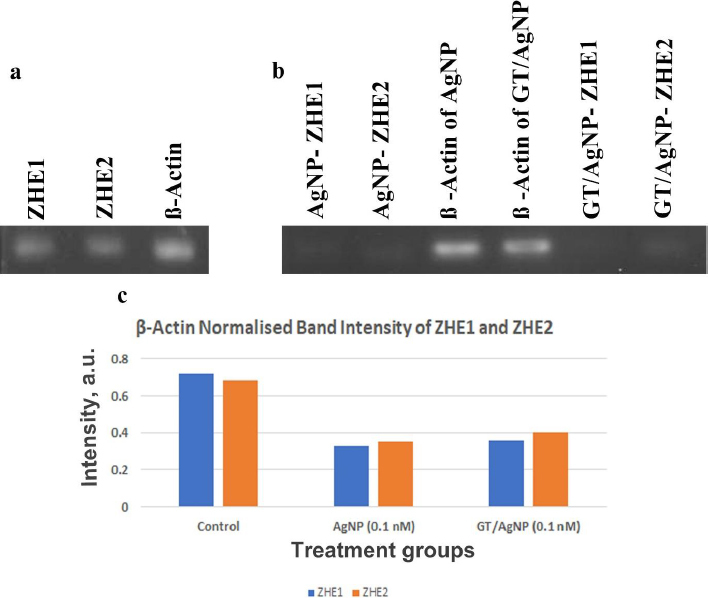
a - Gene expression level of ZHE1, ZHE2 and β actin for control embryos, b - gene expression level of ZHE1, ZHE2 and β actin for embryos treated with 0.2 nM concentration of AgNP and GT/AgNP, respectively, c - β-Actin Normalized Band Intensity of ZHE1 and ZHE2 for control and embryos treated with 0.2 nM concentration of AgNP and GT/AgNP

The gene expression study of ZHE1 and ZHE2 in zebrafish embryos was done with reverse transcriptase PCR (RT-PCR) (Figures 7a and 7b). Beta-actin was used as a housekeeping gene where the ZHE1 was upregulated and the ZHE2 gene was downregulated in the control sample. Thus, from the results, the ZHE2 gene is relatively highly expressed in AgNP and GT/AgNP-treated embryos. ZHE1 is downregulated in AgNP and GT/AgNP-treated zebrafish embryos. Figure 7 shows the differential expression of genes. Figure 7c shows that the GT/AgNP expressed higher levels of ZHE1 and ZHE2 compared to AgNP-treated embryos.

## Discussion

Nanoparticles are profoundly used in different fields of biomedicine [21-23], targeted drug delivery [24, 25], biosensing [26], antimicrobial agents [27,28], imaging [29,30] *etc.* and special mention is warranted for metal and metal oxide nanoparticles. Gold and silver nanoparticles have been used in biomedical fields and theranostics [31]. Among metal nanoparticles, AgNPs are the most commonly used because they are used in various consumer products due to their fungicidal, antiviral, bactericidal and antitumour activity. AgNPs can easily transit the biological membrane and enter the cells, causing toxicity at different levels. Different causes of toxicity exist, such as concentration, shape, size, coating, aggregation, surface charge and the synthesis route, like chemical, biological or physical methods [32,33]. AgNPs cause toxicity by releasing Ag^+^, which accelerates biological membrane damage, ROS production, mitochondrial dysregulation, DNA damage, protein oxidation, and finally inhibits cell proliferation [34]. Worldwide, green tea (*Camellia sinensis*) is one of the common and favourite beverages. Catechins, which belong to the polyphenol family, are the main component of green tea. In total catechins, *Epigallocatechin gallate* accounts for about 50 % and is one of the major polyphenolic compounds in the dried green tea extracts. Consumption of green tea has more beneficial effects on human disease. Cytotoxicity testing was done with A549 cells with green tea extract at various concentrations from 200 to 1000 μg/ml, in which the *IC*_50_ was found to be 500 μM [35]. In this study, we synthesized the silver nanoparticles using trisodium citrate (AgNPs) and green tea extract (GT/AgNPs) and characterized them. The data in Figure S1 in Supplementary Material clearly shows the change in colour of the GT/AgNPs, indicating the successful synthesis. Figure S2 shows typical absorption maxima for AgNPs and GT/AgNPs at 429 nm and 434 nm, respectively. Figure S3 shows the typical hydrodynamic diameter of AgNPs and GT/AgNPs at 65.39 nm and 106.14 nm, respectively, with highly stable values for zeta potential for both nanoparticles. As indicated in Figure S4, the FTIR peaks also supported the synthesis of AgNPs and GT/AgNPs. Altogether, the characterizations supported the notion that the silver nanoparticles were successfully synthesized chemically and by the green route. To examine the effect of AgNPs and GT/AgNPs on the growth inhibition of *E. coli*, we have done an antibacterial assay. Figure S5 shows that neither AgNPs and GT/AgNPs nor the reducing agents trisodium citrate and green tea extract could exert any bacterial killing effect. This showed that our selected dose was very low to elicit any anti-bacterial activity. A step ahead, we explored the toxicity of chemically synthesized AgNPs and green tea extract assisted silver nanoparticles (GT/AgNPs) at low concentrations in cancer cells and normal cells. It was evident that GT/AgNP at 0.2 nM concentration killed the A549 cells at a higher rate compared to other concentrations. MTT assay was performed in a normal cell line (L132 cells), showing that none of the nanoparticles could kill the normal cells (Figure 1). These data suggested a specificity of the GT/AgNPs towards cancer cell killing, and the killing rate was higher than that of chemically synthesized AgNPs. The possible reason behind this result may be due to the leaky nature of the cancer cell membranes, which allows higher uptake of silver nanoparticles compared to normal cells with intact membranes. Thus, the silver ions reaching the organelles inside the cancer cells at higher amounts elicit higher cell killing. The nanoparticles are disposed of in aquatic systems and aquatic flora and fauna are affected. Further, to explore the effect of both AgNPs and GT/AgNPs in the aqueous environment, we utilized the zebrafish embryos. Due to their small size, optical embryo transparency, high reproducibility, rapid development, and fast hatchability, zebrafish embryos act as a very apt model for studying the toxicity of nanoparticles. Zebrafish embryos act as a good model system to study aquatic animal toxicity. Mashjoor et al. made a study with the chemically synthesized AgNPs, which showed that the lethal concentration 50 (LC_50_) for adult fishes after 96 h was found to be 0.788 mg/l and at a high dose of 3 mg/l, the mortality rate was 100 % [36]. In another study, the fate and effect of AgNP were evaluated with *Danio rerio* larvae, which were exposed to different concentrations of AgNPs. The impairment of growth was not significant, but the AgNPs accumulated in the liver, intestine, and inside the liver blood vessels and possessed changes in 65 gene expression [37]. Zebrafish exposure to AgNPs coated with PVP showed a lethal concentration (LC_50_) of 84 and 25 μg/l after 48 h incubation. The operculum movement rate was enhanced, and there was an elevation in surface respiration after treatment with AgNPs, which indicated respiratory toxicity [38]. Qiang *et al.* [4] studied the growth and uptake of AgNPs in zebrafish with 4 nm size silver nanoparticles at concentrations above 0.963 mg/l [4]. Remarkable *in vivo* uptake and delay in the absorption of yolk sac was observed and at a concentration of about 1.925 mg/l, the body length was reduced compared to the control embryos. Further, toxicity was not observed after treatment with the 10 nm sized AgNPs [4]. In this study, chemically synthesized AgNPs were more toxic to the zebrafish embryos, even at lower concentrations. In the embryos treated with AgNPs, at a higher concentration of 0.5nM, the mortality rate was about 100 % and for embryos treated with GT/AgNPs, the hatchability was 78 % at the same dose. Moreover, many abnormalities were observed in the zebrafish embryos after treatment with chemically synthesized AgNPs, which was not seen for the GT/AgNPs-treated embryos at the same doses. This observation indicated that GT/AgNPs were far more biocompatible compared to chemically synthesized AgNPs. To rule out the gene level effect manifested by the decreased hatchability percentage of embryos, gene expression studies was performed. ZHE1 and ZHE2 were the prime genes involved in the hatching of zebrafish embryos. We observed a differential gene expression of these two genes in the embryos treated with chemically synthesized and green synthesized zebra fish embryos. There was a decrease in both the gene expression for AgNP and GT/AgNP treated embryos, but the decrease was more pronounced for the chemically synthesized AgNP treated embryos.

## Conclusions

The lower toxic species of silver nanoparticles concentration is studied in in vitro and in vivo. Our in vitro data suggested that the GT/AgNPs could kill the cancer cells more efficiently than AgNPs, whereas no effect was elicited for normal cells. Further studies are needed to determine the mechanism of specific cancer cell killing. Gene expression of ZHE1 and ZHE2, which are prime genes responsible for hatching zebrafish embryos, was also studied. The data suggest that the ZHE1 gene is downregulated in AgNP and GT/AgNP treated embryos, which could be the reason for the non-hatchability of zebrafish embryos. The data clearly demonstrated that green tea-mediated synthesis (GT/AgNP) is less toxic and, as such, can be a promising option for various applications in both environmental and industrial sectors. However, further research is essential to explore the cellular mechanisms undergone when exposed to low doses of silver nanoparticles.

## Supplementary material

Additional data are available at https://pub.iapchem.org/ojs/index.php/admet/article/view/2102, or from the corresponding author on request.


