# Supplementary material for: Beneficial effects of bioinspired silver nanoparticles on zebrafish embryos including a gene expression study
Source: ADMET DMPK. 2024 Jan 1;12(1):177–92. doi: 10.5599/admet.2102 (PMC10974822; doi:10.5599/admet.2102)
Supplement: Supplementary file 1 [file ADMET-12-2102-S1.pdf]

ADMET & DMPK 12(1) (2024) S1-S3

Open Access : ISSN : 1848-7718

http://www.pub.iapchem.org/ojs/index.php/admet/index

Supplementary material to

Beneficial effects of bioinspired silver nanoparticles on zebrafish embryos including a gene expression study

Sakthi Devi R, Agnishwar Girigoswami, Shanmugaraja Meenakshi, Balasubramanian Deepika, Karthick Harini, Pemula Gowtham, Pragya Pallavi and Koyeli Girigoswami

Medical Bionanotechnology, Faculty of Allied Health Sciences, Chettinad Hospital & Research Institute (CHRI), Chettinad Academy of Research and Education (CARE), Kelambakkam, Chennai-603 103, India

ADMET & DMPK 12(1) (2024), 177-192; https://doi.org/10.5599/admet.2102

Time, min	Change in colour		Time, min	Change in colour	
	beaker 1	beaker 2		beaker 1	beaker 2
0	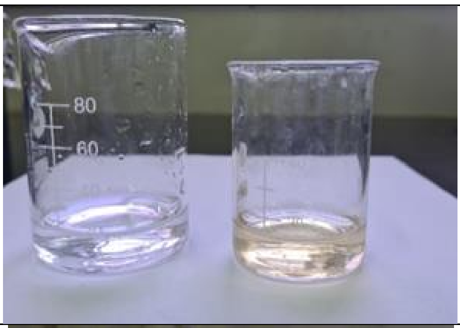		4	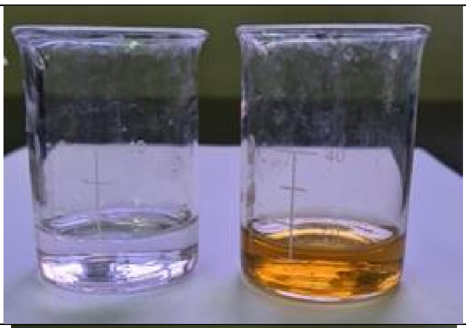	
1	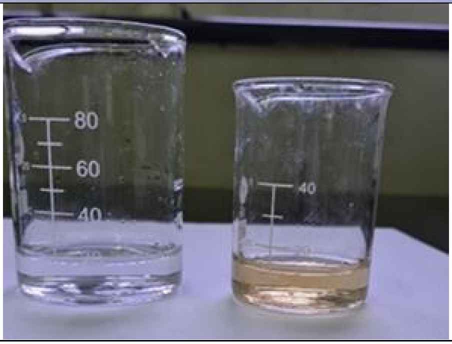		6	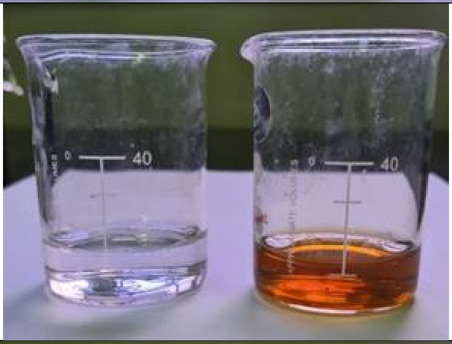	
3	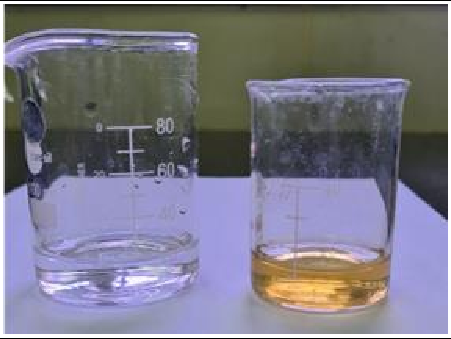		11	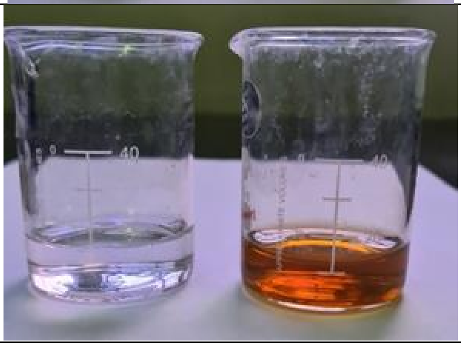	

Figure S1. The green synthesis of silver nanoparticles was observed by change in colour at different time intervals (0, 1, 3, 4, 6 and 11 min) after addition of green tea extract. Beaker 1 contains silver nitrate; beaker 2 contains silver nitrate and green tea extract

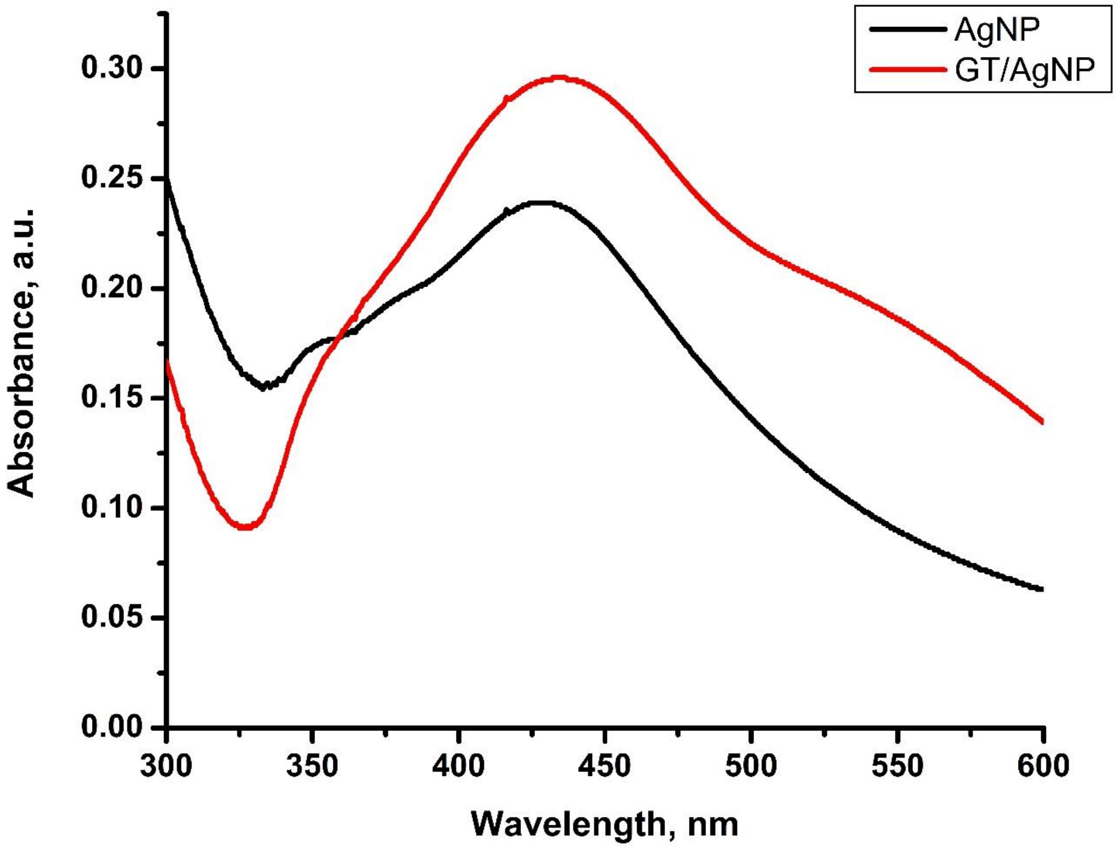

Figure S2. The UV-Visible absorbance spectra for AgNPs and GT/AgNPs

<table>
<tr><th>Particles</th><th>Hydrodynamic diameter</th><th>Surface Charge</th></tr>
<tr><td>AgNP</td><td>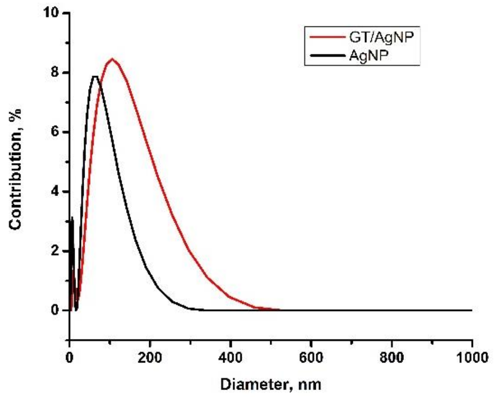

AgNP- 65.39 nm
GT/AgNP- 106.14 nm</td><td>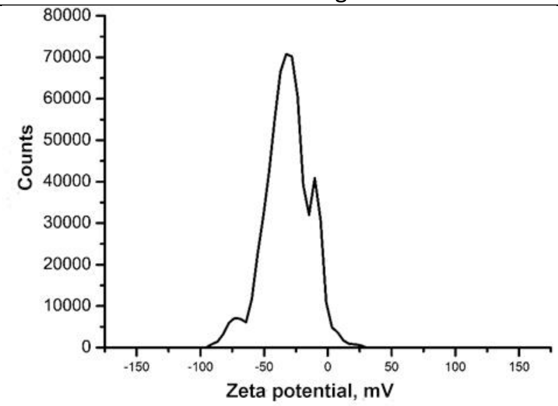

AgNP = -25.1 mV</td></tr>
<tr><td>GT/AgNP</td><td></td><td>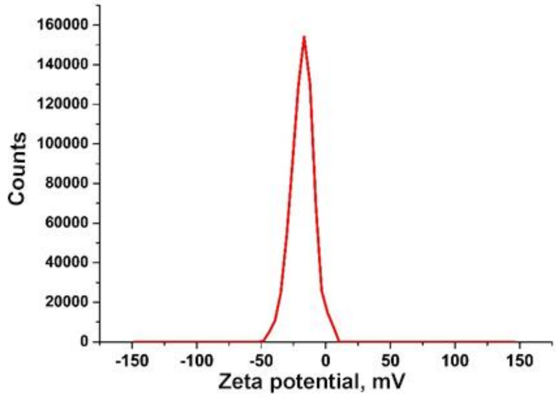

GT/AgNP = -16.9 mV</td></tr>
</table>

Figure S3. The hydrodynamic diameter and the zeta potential of as synthesized AgNPs and GT/AgNPs

CC BY

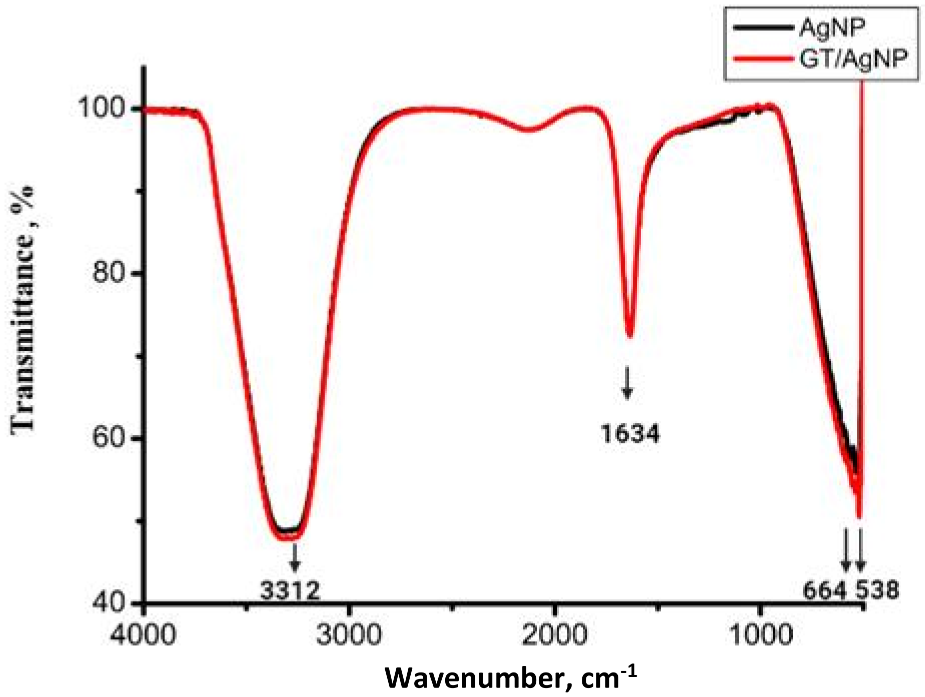

Figure S4. The FTIR spectra for AgNPs and GT/AgNPs.

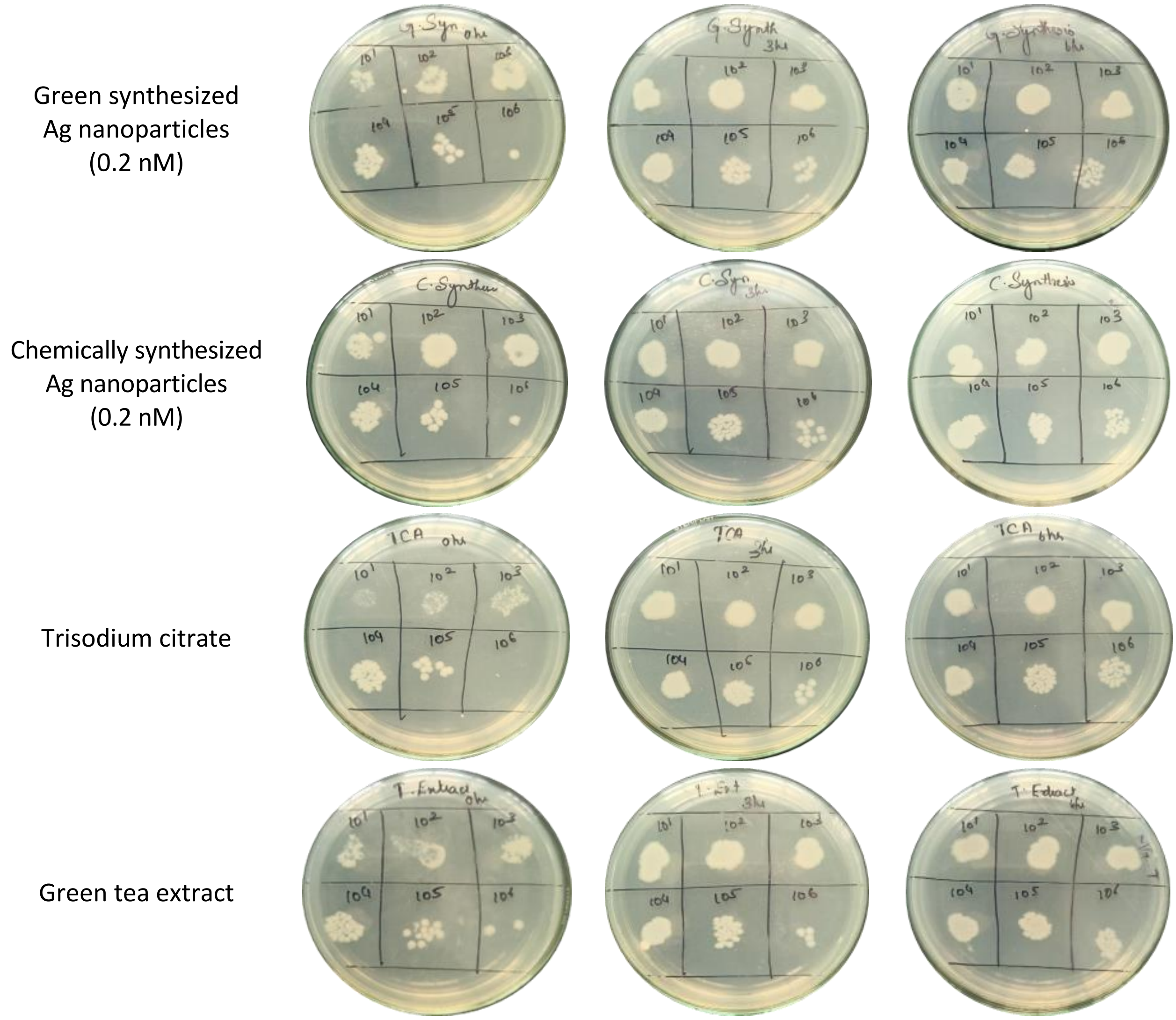

Figure S5. The growth of E. coli at different dilutions (10^1, 10^2, 10^3, 10^4, 10^5, 10^6) after teatment with GT/AgNPs, Ag NPs, trisodium citrate and green tea extract
