# Supplementary material for: Beneficial effects of bioinspired silver nanoparticles on zebrafish embryos including a gene expression study
Source: ADMET DMPK. 2024 Jan 1;12(1):177–92. doi: 10.5599/admet.2102 (PMC10974822; doi:10.5599/admet.2102)
Supplement: Supplementary file 2 [file ADMET-12-2102-S1.docx]

*ADMET & DMPK 12(1) (2024) S1-S3*

*
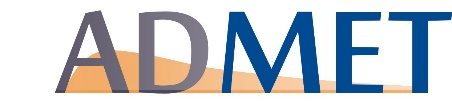
***Open Access : ISSN : 1848-7718**[***http://www.pub.iapchem.org/ojs/index.php/admet/index***](http://www.pub.iapchem.org/ojs/index.php/admet/index)

Supplementary material to

Beneficial effects of bioinspired silver nanoparticles on zebrafish embryos including a gene expression study

Sakthi Devi R, Agnishwar Girigoswami, Shanmugaraja Meenakshi, Balasubramanian Deepika, Karthick Harini, Pemula Gowtham, Pragya Pallavi and Koyeli Girigoswami

Medical Bionanotechnology, Faculty of Allied Health Sciences, Chettinad Hospital & Research Institute (CHRI), Chettinad Academy of Research and Education (CARE), Kelambakkam, Chennai-603 103, India

ADMET & DMPK 12(1) (2024), 177-192; <https://doi.org/10.5599/admet.2102>

| Time, min | Change in colour | Time, min | Change in colour |
| --- | --- | --- | --- |
|  | beaker 1 beaker 2 |  | beaker 1 beaker 2 |
| 0 | 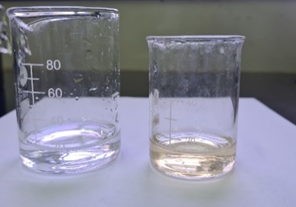 | 4 | 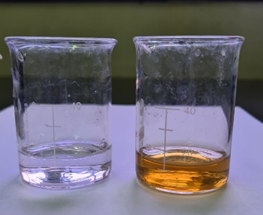 |
| 1 | 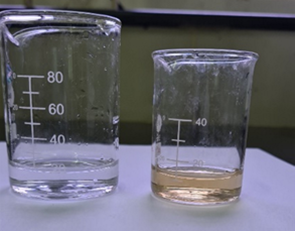 | 6 | 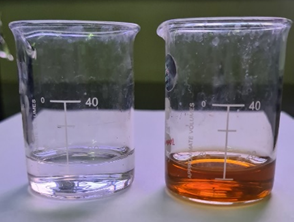 |
| 3 | 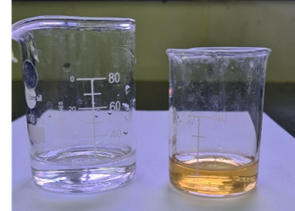 | 11 | 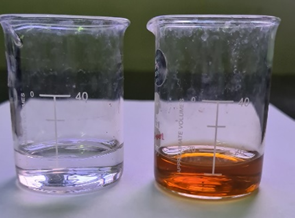 |

**Figure S1.** The green synthesis of silver nanoparticles was observed by change in colour at different time intervals (0, 1, 3, 4, 6 and 11 min) after addition of green tea extract. Beaker 1 contains silver nitrate; beaker 2 contains silver nitrate and green tea extract


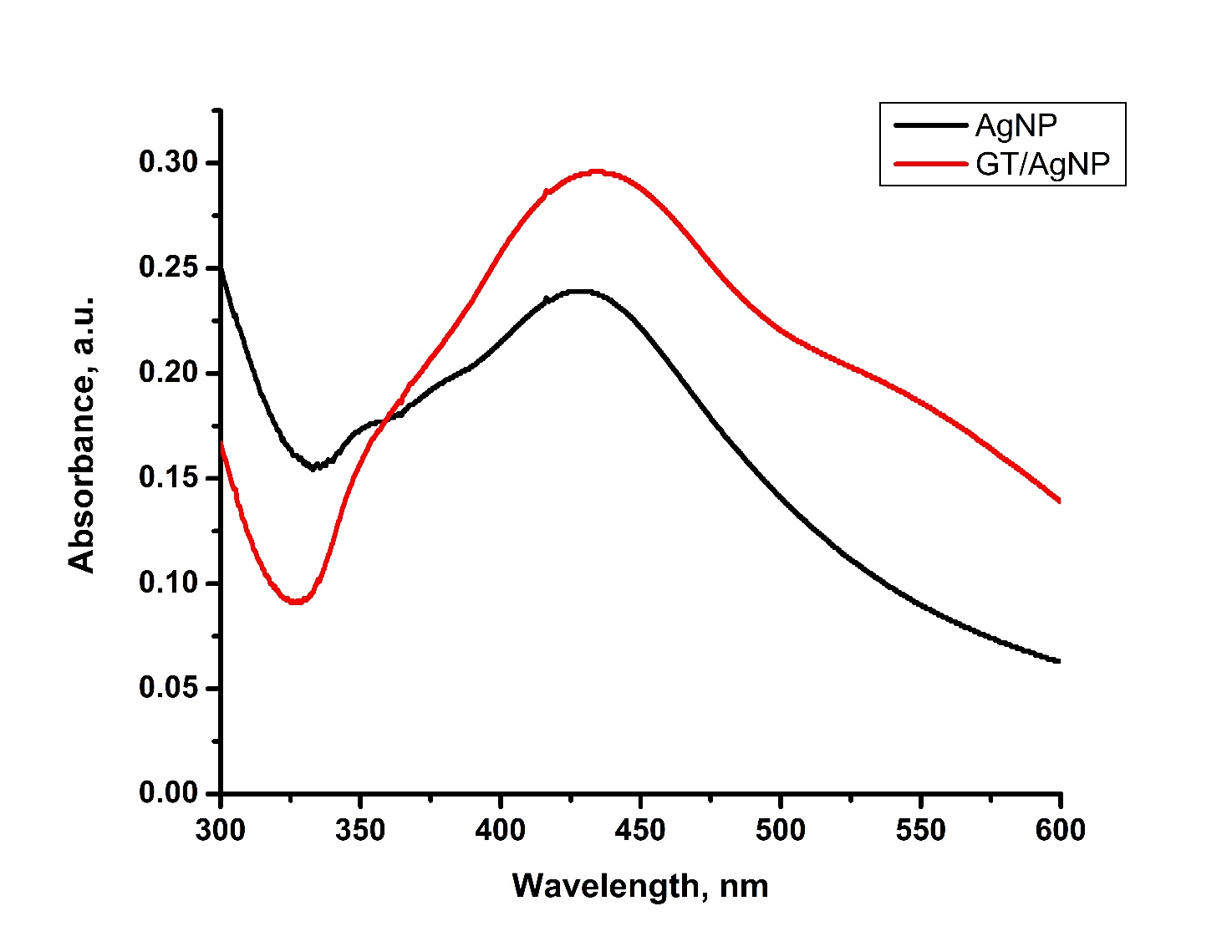


**Figure S2.** The UV-Visible absorbance spectra for AgNPs and GT/AgNPs

| Particles | Hydrodynamic diameter | Surface Charge |
| --- | --- | --- |
| AgNP | 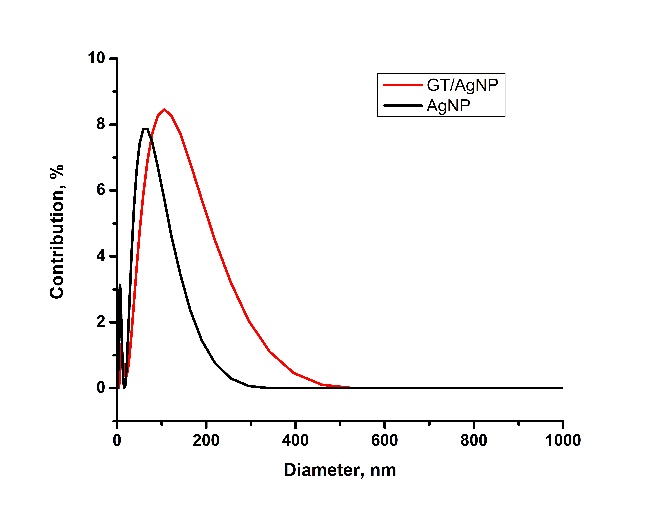  AgNP- 65.39 nm  GT/AgNP- 106.14 nm | 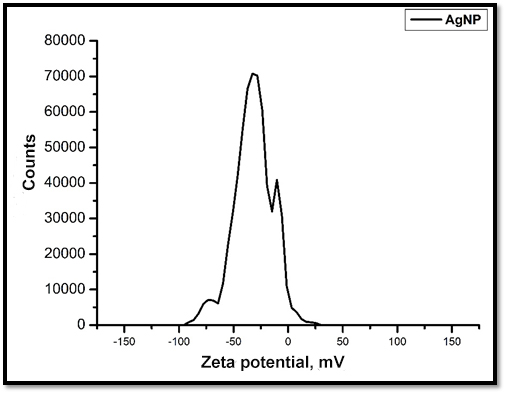  AgNP = -25.1 mV |
| GT/AgNP |  | 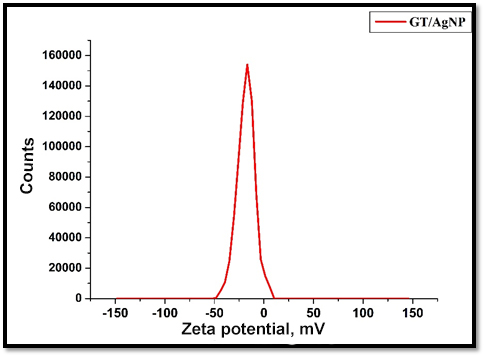  GT/AgNP = -16.9 mV |

**Figure S3.** The hydrodynamic diameter and the zeta potential of as synthesized AgNPs and GT/AgNPs


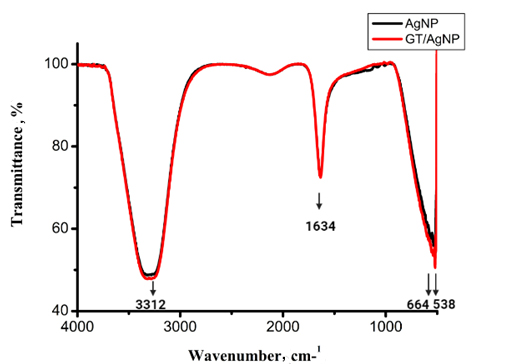


**Wavenumber, cm^-1^**

**Figure S4.** The FTIR spectra for AgNPs and GT/AgNPs.

| Green synthesized  Ag nanoparticles  (0.2 nM) | 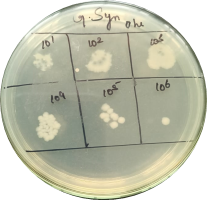 | 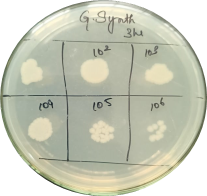 | 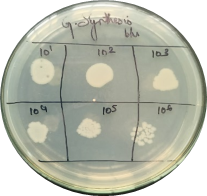 |
| --- | --- | --- | --- |
| Chemically synthesized  Ag nanoparticles  (0.2 nM) | 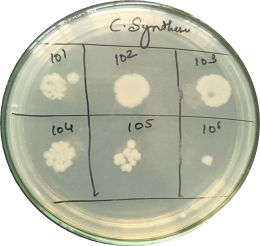 | 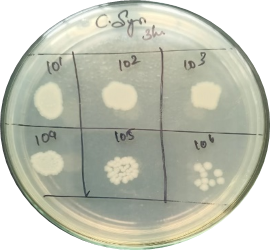 | 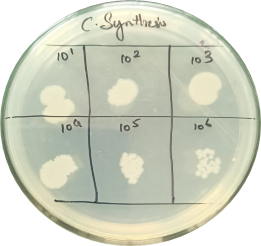 |
| Trisodium citrate | 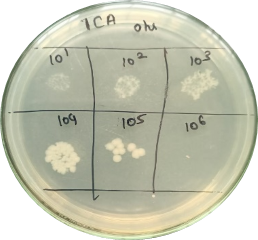 | 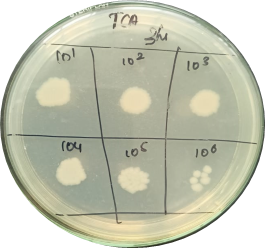 | 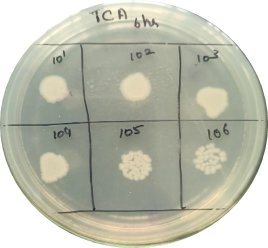 |
| Green tea extract | 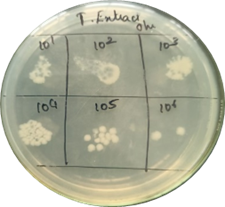 | 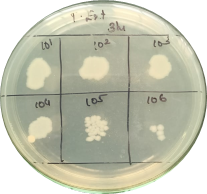 | 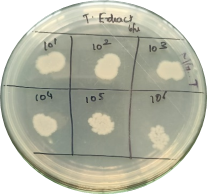 |

**Figure S5.** The growth of E. coli at different dilutions (10^1^, 10^2^, 10^3^, 10^4^, 10^5^, 10^6^) after teatment with GT/AgNPs, Ag NPs, trisodium citrate and green tea extract
